# Methodological Framework to Evaluate Entomopathogenic Fungi and Rhizobial Co-Inoculation Effects on Plant Growth and Root Morphology

**DOI:** 10.3390/plants15142141

**Published:** 2026-07-10

**Authors:** Tamiris dos Santos Lopes, Emily Mesquita, Joana da Rocha Matos, Thais Almeida Correa, Tadeu Augusto van Tol de Castro, Andrés Calderín Garcia, João Luiz Lopes Monteiro Neto, Gabriela Cavalcanti Alves, Jerri Édson Zilli, Isabele da Costa Angelo, Wendell Marcelo de Souza Perinotto, Vânia Rita Elias Pinheiro Bittencourt, Patrícia Silva Golo

**Affiliations:** 1 Postgraduate Program in Plant Health and Applied Biotechnology, Institute of Biological and Health Sciences, Federal Rural University of Rio de Janeiro, BR 465, km 07, Seropédica 23897-035, RJ, Brazil; tamirisdslopes@gmail.com (T.d.S.L.); thaisalmeida_tac@yahoo.com.br (T.A.C.); 2 Veterinary Institute, Federal Rural University of Rio de Janeiro, BR 465, km 07, Seropédica 23897-035, RJ, Brazil; emilymesquita@ufrrj.br (E.M.); rochajoana51@gmail.com (J.d.R.M.); 3 Laboratory of Soil Biological Chemistry, Department of Soils, Institute of Agronomy, Federal Rural University of Rio de Janeiro, BR 465, km 07, Seropédica 23897-035, RJ, Brazil; tadeuvantol@gmail.com (T.A.v.T.d.C.); cg.andres@gmail.com (A.C.G.); 4 Department of Plant Science, Agronomy Institute, Federal University of Roraima, BR 174, Km 12, s/n—Zona Rural, Boa Vista 69310-000, RR, Brazil; joao.luiz@ufrr.br; 5 Embrapa Agrobiologia, BR 465, km 07, Seropédica 23891-000, RJ, Brazil; gabcalves@gmail.com (G.C.A.); jerri.zilli@embrapa.br (J.É.Z.); 6 Department of Epidemiology and Public Health, Veterinary Institute, Federal Rural University of Rio de Janeiro, BR 465, km 07, Seropédica 23897-035, RJ, Brazil; isabeleangelo@yahoo.com.br (I.d.C.A.); wendellperinotto@ufrrj.br (W.M.d.S.P.); 7 Department of Animal Parasitology, Veterinary Institute, Federal Rural University of Rio de Janeiro, BR 465, km 07, Seropédica 23897-035, RJ, Brazil

**Keywords:** *Metarhizium*, *Bradyrhizobium*, soybean, plant–microbe interactions, bioinput

## Abstract

Entomopathogenic fungi are increasingly recognized as multifunctional bioinputs, but robust methodological approaches are needed to evaluate their compatibility with established microbial inoculants and their effects on plant performance. Our study proposes an integrative framework to assess *Metarhizium–Bradyrhizobium* interactions in soybean (*Glycine max*), combining laboratory compatibility screening with greenhouse assessment. First, *Metarhizium anisopliae* LCM S04 and *Metarhizium brunneum* LCM S11 were tested against *Bradyrhizobium diazoefficiens* BR 85 (=SEMIA 5080) and *Bradyrhizobium japonicum* BR 86 (=SEMIA 5079) using dual-culture assays in a medium that supported the growth of both fungal and bacterial partners, allowing direct evaluation of intermicrobial compatibility. Subsequently, the microorganisms were evaluated in soybean under greenhouse conditions through direct seed inoculation. Each seed received bacterial culture, fungal suspension, or both (including absolute and nitrogen-fertilized controls). Plants were maintained under controlled conditions for 47 days. Plant height, leaf production, branching, nodulation, biomass, and root morphology were assessed. No inhibition zones were observed in vitro, and co-inoculation did not impair nodulation. The combination BR 86 + LCM S04 improved fine root number in soybean. This framework provides a reproducible approach for evaluating microbial compatibility and functional bioinput benefits and can be adapted to crops other than soybean.

## 1. Introduction

Entomopathogenic fungi (EPF) are key regulators of insect population dynamics in agricultural ecosystems [1]. In the Brazilian biopesticide market, among invertebrate fungal pathogens, the genera *Beauveria* (Hypocreales: Cordycipitaceae) and *Metarhizium* (Hypocreales: Clavicipitaceae) account for over 70% of all commercially available fungal biocontrol agents in the country [2,3]. Beyond their role as insect pathogens, *Metarhizium* species are increasingly recognized as multifunctional microorganisms capable of colonizing the rhizosphere and plant tissues as root symbionts and endophytes [4,5,6,7]. This intimate association with plants extends their ecological role beyond arthropod control, enabling them to participate in tripartite interactions among soil, plants, and insect pests [8,9]. As a consequence, EPF can contribute to plant health not only through pest suppression but also by enhancing plant performance and increasing tolerance to biotic and abiotic stresses, including phytopathogens and salinity [10,11,12].

The effectiveness of isolates like *Metarhizium brunneum*, *Metarhizium anisopliae*, and *Metarhizium robertsii* is often linked to their rhizosphere competency [13,14], particularly when applied as seed treatments or through diverse inoculation strategies [15,16,17]. Despite growing recognition of these benefits [18,19], information regarding the association of *Metarhizium* spp. with crops in Brazil is in its early stages [7]. Elucidating these symbiotic interactions within the rhizosphere is crucial for advancing sustainable agriculture. This is because the rhizosphere is a critical hub for intricate plant–microbe and microbe–microbe interactions that are responsible for nutrient cycling, ecosystem stability, and carbon sequestration [20,21].

As the world’s leading oilseed crop, soybean (*Glycine max*) relies extensively on *Bradyrhizobium* spp. bacteria to drive Biological Nitrogen Fixation (BNF). This symbiotic process is a widely used sustainable alternative, meeting the high nitrogen demand of soybean, which exceeds 80 kg of nitrogen ton of grain^−1^ [22]. In South America, annual inoculation of over 40 million hectares with *Bradyrhizobium* enables the fixation of up to 10 million tons of nitrogen year^−1^, averaging over 200 kg ha^−1^ harvest^−1^ [23,24,25]. Nevertheless, the success of this symbiosis depends not only on the competitiveness of rhizobial strains but also on their compatibility with the surrounding soil microbiota [26].

In this context, understanding how entomopathogenic fungi interact with rhizobial inoculants has become increasingly relevant. Although endophytic colonization by EPF can activate systemic plant defenses and enhance resistance and tolerance to insect herbivores [27,28], whether these plant responses influence interactions with rhizobial inoculants remains poorly understood. Combining biological pest control with biological nitrogen fixation therefore represents a promising strategy for developing multifunctional microbial consortia. However, despite growing interest in microbial consortia, studies evaluating the compatibility between EPF and nitrogen-fixing bacteria, particularly *Bradyrhizobium* spp., remain scarce.

Recent findings have demonstrated that the biological performance of *Metarhizium* in soybean is strongly isolate-dependent [29]. When screening native Uruguayan isolates for compatibility with commercial *Bradyrhizobium elkanii* strains (U1301 and U1302), the authors reported in vitro inhibitory interactions of specific isolates. These included seven *Metarhizium lepidiotae* isolates with strong inhibitory effects, as well as *M. robertsii* ILB440 and *Metarhizium frigidum* ILB185 with moderate inhibition, suggesting potential direct antagonism under laboratory conditions. However, these inhibitory effects were not observed in planta studies, where co-inoculation showed no interference with nodulation or plant performance. Additionally, although that study [29] demonstrated the compatibility of selected *Metarhizium* isolates with *Bradyrhizobium* in soybean, important gaps remain regarding standardized compatibility assessments and the effects of co-inoculation on root system architecture.

Selecting microorganisms for new commercial formulations requires a multifaceted evaluation. In the context of microbial consortia, success will rely on the biological compatibility between inoculants and the assurance of their viability [30]. Thus, there is a need for methodological guides that enable a comprehensive evaluation of plant–microbe interactions.

To our knowledge, this is the first study to explore the compatibility and co-inoculation of EPF with specific *Bradyrhizobium* strains, which are essential in Brazilian soybean production. By analyzing the in vitro and greenhouse interactions between the officially recommended strains *B. diazoefficiens* BR 85 (CPAC 7; SEMIA 5080) and *B. japonicum* BR 86 (CPAC 15; SEMIA 5079) and two soil-derived fungal isolates (*M. anisopliae* LCM S04 and *M. brunneum* LCM S11), we propose a standardized framework to evaluate microbial compatibility and plant growth promotion. This methodology is designed to be broadly applicable, extending beyond soybean to other leguminous crops where seed inoculation with entomopathogenic fungi has already shown promising results [18,31], and can be adapted for multiple fungal genera and various plant species. Furthermore, the present study aims to investigate how such co-inoculations impact root morphology and the maintenance of rhizobial symbiosis.

## 2. Results

### 2.1. In Vitro Compatibility Test

**Figure 1:**
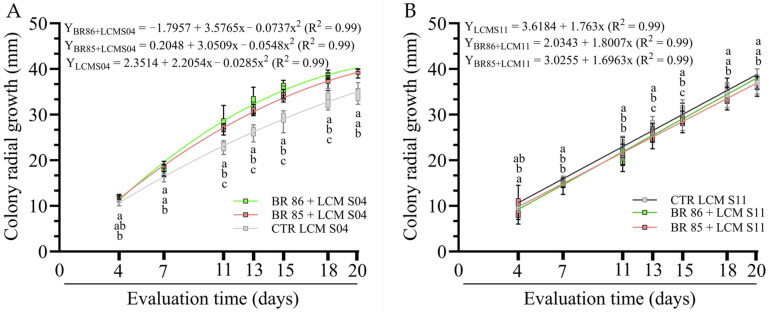
Colony radial growth of *Metarhizium* isolates in dual culture with *Bradyrhizobium* strains evaluated at 4, 7, 11, 13, 15, 18, and 20 days after incubation (DAI): (**A**) isolate LCM S04 (*Metarhizium anisopliae*); and (**B**) isolate LCM S11 (*Metarhizium brunneum*), both confronted with strains BR 85 (*Bradyrhizobium diazoefficiens*) and BR 86 (*Bradyrhizobium japonicum*). Different letters in each evaluation day (vertical) indicate significant differences among the groups according to Tukey’s test (*p* < 0.05).

Compatibility was evaluated using two complementary criteria: (i) the absence of visible antagonistic interactions, such as inhibition zones or growth suppression at the fungus–bacterium interface, and (ii) the maintenance of fungal colony development throughout the incubation period. Fungal monocultures (LCM S04 and LCM S11) served as controls, providing a baseline for radial growth and allowing a direct comparison with the corresponding co-culture treatments. No inhibition zones or signs of antagonism were observed in any fungal–bacterial combination evaluated (Figure 1, Figures S1 and S2). Colony growth was maintained in all treatments, indicating that the selected PDA-based dual-culture system was suitable for simultaneously supporting fungal and bacterial development and for detecting potential negative interactions between the microorganisms.

Regression analyses confirmed a significant effect of incubation time on colony expansion for both fungal isolates. For LCM S04, co-cultivation with either BR 85 or BR 86 resulted in greater radial growth than the fungal control throughout most of the evaluation period (Figure 1A). In contrast, LCM S11 exhibited slightly lower radial growth in dual culture than in monoculture (Figure 1B). Despite the reduced growth, no inhibition or antagonistic zones were observed. The regression model adjustment for all treatments exhibited high coefficients of determination (R^2^ = 0.99), confirming a linear relationship between the radial growth of LCM S11 and incubation time.

The assay successfully distinguished between neutral, stimulatory, and mildly suppressive interactions while confirming the absence of antagonism.

### 2.2. Plant Inoculation Test

#### 2.2.1. Plant Growth and Development

The experimental design included two reference controls with distinct purposes: an absolute control (CTR), representing plant development in the absence of microbial inoculation, and a nitrogen-fertilized control (NIT), representing plant growth under non-limiting nitrogen availability. Single inoculations of *Bradyrhizobium* strains and *Metarhizium* isolates were also included, allowing the individual contribution of each microorganism to be distinguished from the effects of co-inoculation.

**Figure 2:**
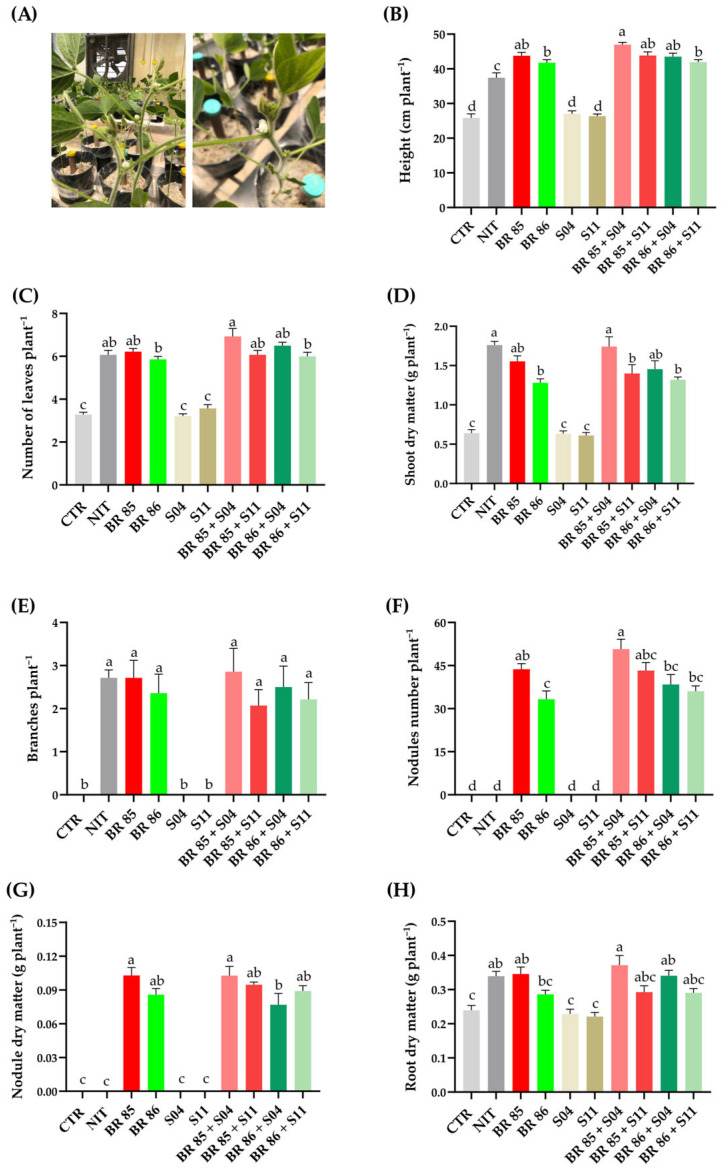
Mean and standard error of agronomic and nodulation variables of soybean (*Glycine max*) plants. (**A**) Representative image of a soybean plant at the time of harvest, showing details of the beginning of flowering at 47 days post-inoculation (DPI); (**B**) Height (cm plant^−1^); (**C**) Number of leaves (plant^−1^); (**D**) Shoot dry matter (g plant^−1^); (**E**) Number of branches (plant^−1^); (**F**) Nodule number (plant^−1^); (**G**) Nodule dry matter (g plant^−1^); and (**H**) Root dry matter (g plant^−1^). (CTR) control; (NIT) nitrogen control; BR 85 (*Bradyrhizobium diazoefficiens*), BR 86 (*Bradyrhizobium japonicum*), LCM S04 (*Metarhizium anisopliae*), LCM S11 (*Metarhizium brunneum*), and co-inoculations (BR 85 + S04, BR 86 + S04, BR 85 + S11, and BR 86 + S11). Means followed by the same letter do not differ significantly from each other after Tukey’s test (*p* < 0.05), n = 7.

Plant height, leaf number, branch emission, and shoot dry matter were used as indicators of overall plant vigor and vegetative development. Across these variables, treatments containing *Bradyrhizobium*, either alone or in combination with *Metarhizium*, generally showed superior performance compared with the uninoculated and fungal-only controls (Figure 2). For example, the presence of *Bradyrhizobium* spp., either alone or in co-inoculation, doubled the number of leaves compared to that in CTR (3.29 ± 0.10) and treatments with LCM S04 (3.21 ± 0.10) and LCM S11 (3.57 ± 0.17). Both LCM S04 and LCM S11 were not different when compared to CTR. Accordingly, bacterial treatments alone were not different from co-inoculated treatments. In this context, the inclusion of fungal monocultures was particularly important for determining whether observed responses resulted from the fungal inoculant itself or from interactions established with rhizobial strains.

Nodulation parameters were considered key indicators of compatibility because successful co-inoculation should not impair the establishment of the rhizobia–legume symbiosis. Therefore, nodule number and nodule dry matter were used as primary screening variables to verify whether the presence of *Metarhizium* interfered with biological nitrogen fixation. Nodulation occurred exclusively in treatments containing *Bradyrhizobium* and was maintained in all co-inoculated groups, demonstrating that fungal inoculation did not disrupt symbiotic establishment. In some combinations, particularly those involving BR 85 and LCM S04, nodulation parameters were further enhanced relative to single bacterial inoculation.

Root dry matter was included as an integrative indicator of belowground plant development and resource allocation. Similar to the agronomic variables, treatments containing *Bradyrhizobium* generally exhibited greater root biomass than the uninoculated and fungal-only controls. The BR 85 + LCM S04 treatment (0.37 ± 0.02 g) achieved the highest accumulation, followed by BR 85 alone (0.34 ± 0.02 g), the BR 86 + LCM S04 co-inoculation (0.34 ± 0.01 g) and NIT (0.33 ± 0.01 g). The CTR, LCM S04 and LCM S11 treatments showed the lowest values, with averages around 0.22 g to 0.24 g.

Together, these results demonstrate how the proposed framework can simultaneously evaluate biological compatibility, preservation of nodulation, and functional effects on plant growth. From a methodological perspective, the most informative variables for initial screening were nodule formation and biomass accumulation, as they directly indicate whether microbial co-inoculation maintains symbiotic performance while providing additional agronomic benefits.

#### 2.2.2. Effects on Root Morphology

**Figure 3:**
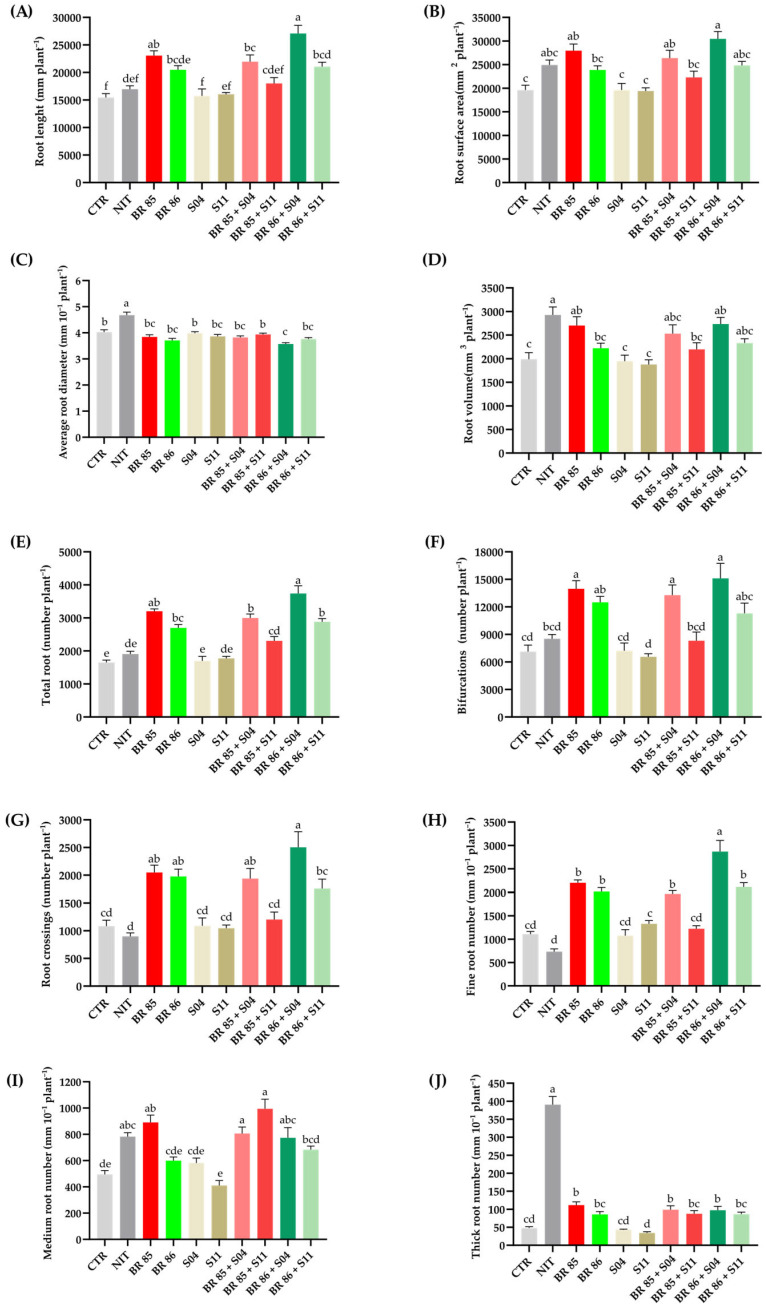
Mean and standard error of root parameters in soybean (*Glycine max*) under the following treatments: control (CTR), nitrogen control (NIT); BR 85 (*Bradyrhizobium diazoefficiens*), BR 86 (*Bradyrhizobium japonicum*), LCM S04 (*Metarhizium anisopliae*), LCM S11 (*Metarhizium brunneum*), and co-inoculations (BR 85 + LCM S04, BR 86 + LCM S04, BR 85 + LCM S11, and BR 86 + LCM S11) at 47 days post-inoculation. (**A**) Root length (mm plant^−1^); (**B**) root surface area (mm^2^ plant^−1^); (**C**) average root diameter (mm root^−1^ plant^−1^); (**D**) root volume (mm^3^ plant^−1^); (**E**) total root number (roots plant^−1^); (**F**) bifurcations plant^−1^; (**G**) root crossings plant^−1^; (**H**) fine root number; (**I**) medium root number; and (**J**) thick root number. Means followed by the same letter do not differ significantly from each other according to Tukey’s test at 5% probability (*p* < 0.05), n = 7.

The root analysis included both structural and architectural descriptors obtained through image-based root phenotyping (Figure 3). Structural variables such as root length, surface area, average diameter, and root volume were selected to characterize overall root system development. In parallel, architectural variables including total root number, bifurcations, crossings, and the abundance of fine, medium, and thick roots were evaluated to detect changes in root complexity and branching patterns that may not be evident through biomass measurements alone.

The experimental design incorporated uninoculated (CTR) and nitrogen-fertilized (NIT) controls, as well as fungal and bacterial monocultures, allowing the individual and combined effects of each microorganism to be distinguished. This approach enabled the identification of root traits specifically associated with co-inoculation rather than with the presence of a single microbial partner.

Overall, treatments containing *Bradyrhizobium* exhibited superior root development compared with the uninoculated and fungal-only controls, confirming the central role of rhizobial symbiosis in soybean root performance. However, several root architecture parameters were further enhanced in specific co-inoculation treatments, particularly those involving *M. anisopliae* LCM S04. For instance, regarding root surface area, the BR 86 + LCM S04 treatment showed the highest value (30,473.5 mm^2^), differing statistically from the CTR (19,605.4 mm^2^) and the treatments that received only fungal inoculation. The former treatment also showed a significant difference compared to BR 86 alone (23,868.7 mm^2^) (Figure 3B). These responses were observed across multiple descriptors, including root length, total root number, bifurcations, crossings, and fine root abundance, indicating that root architecture analysis can reveal functional benefits that are not always detected by conventional agronomic measurements alone.

**Figure 4:**
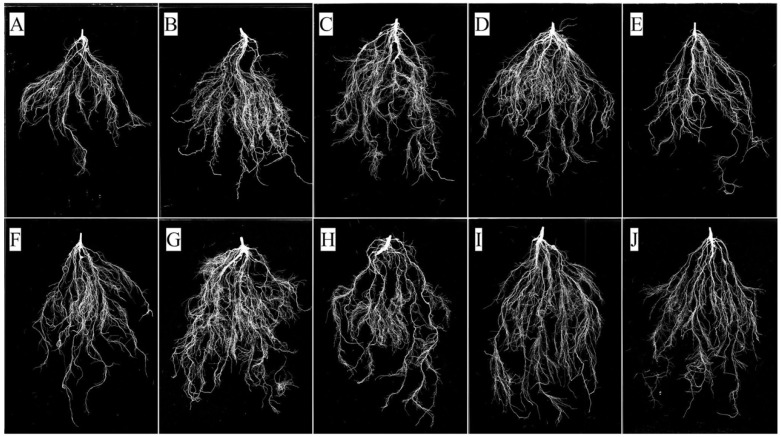
Representative root images of soybean (*Glycine max*) (47 days post-inoculation) obtained via a WinRHIZO scanner under different treatments: (**A**) control (CTR); (**B**) nitrogen control (NIT); (**C**) BR 85 (*Bradyrhizobium diazoefficiens*); (**D**) BR 86 (*Bradyrhizobium japonicum*); (**E**) LCM S04 (*Metarhizium anisopliae*); (**F**) LCM S11 (*Metarhizium brunneum*); (**G**) BR 85 + LCM S04; (**H**) BR 85 + LCM S11; (**I**) BR 86 + LCM S04; and (**J**) BR 86 + LCM S11.

The inclusion of parameters such as root length, surface area, root volume, total root number, bifurcations, and root diameter enables a comprehensive characterization of both root size and architectural complexity, generating information that is suggested to be associated with soil exploration and resource acquisition. Representative root images obtained at harvest (Figure 4) qualitatively illustrate the differences in root system development among treatments and complement the quantitative measurements generated through image analysis, particularly regarding root density and branching patterns.

#### 2.2.3. Qualitative Detection of *Metarhizium* spp. in the Substrate

Substrate samples were examined to verify the presence and persistence of EPF throughout the experimental period. This assessment was included because the establishment and maintenance of the fungal inoculant in the rhizosphere are important prerequisites for interpreting plant growth responses and potential plant–fungus interactions.

The qualitative detection approach was designed to confirm whether the fungal isolates remained viable in the substrate until the end of the experiment. Control treatments (CTR and NIT) and rhizobial-only treatments served as negative controls, allowing the differentiation between naturally occurring contamination and the persistence of the inoculated fungi. Typical *Metarhizium* colonies were not detected in any negative-control treatment, whereas characteristic green conidial colonies were consistently recovered from substrate samples collected from *Metarhizium*-inoculated groups (Figure S3).

## 3. Discussion

The utilization of multifunctional microorganisms represents an innovative strategy for the sustainable intensification of agricultural systems. In this context, the ecological roles of *Metarhizium* spp. have been redefined beyond biological insect control, highlighting their competence as plant growth promoters and endophytes [14,31,32]. Concurrently, the use of *Bradyrhizobium* spp. remains well-established due to its efficiency in biological nitrogen fixation and its role in promoting the development of legumes [24]. Considering that the successful integration of these microorganisms requires the absence of antagonism, this study proposes a methodological baseline for assessing the compatibility between *Bradyrhizobium* spp. and *Metarhizium* spp., both in vitro and in planta, addressing these two main approaches and providing a framework that can be adapted to other crops, fungal species, and plant–microbe interaction systems.

In the present study, the dual-culture assay was incorporated as the first screening stage of the proposed framework because successful microbial consortia require, at a minimum, the absence of direct antagonistic interactions between their components. The use of PDA as the confrontation medium was selected because it is inexpensive, highly reproducible, and widely adopted for fungal cultivation [33]. Preliminary observations confirmed that both *Bradyrhizobium* strains were also capable of growing on this medium, allowing the simultaneous evaluation of fungal and bacterial development under standardized conditions.

From a methodological perspective, the primary objective of this assay was not to identify the treatment with the greatest radial growth, but rather to determine whether fungal development was impaired in the presence of rhizobia. For this reason, fungal monocultures were included as reference controls, providing a baseline against which all co-culture treatments could be compared. The absence of inhibition zones and the maintenance of colony expansion throughout the incubation period suggested that none of the microbial combinations exhibited antagonistic behavior under the conditions tested. Such information is relevant since incompatible combinations can be discarded before proceeding to greenhouse or field evaluations.

In the present study, the response of LCM S04 in dual culture differed from that of LCM S11, illustrating that compatible microorganisms do not necessarily respond in the same manner. While some combinations may stimulate fungal development, others may produce neutral or mildly suppressive effects. Accordingly, radial growth measurements provided a practical and quantitative indicator for classifying microbial interactions beyond a simple compatible/incompatible dichotomy.

Here, the greenhouse phase of the proposed framework was designed to determine whether microbial combinations previously classified as compatible in vitro maintain their functionality under plant-associated conditions. To achieve this, the experimental design incorporated complementary controls, including uninoculated plants (CTR), a nitrogen-fertilized control (NIT), and fungal and bacterial monocultures. This approach allows researchers to distinguish the individual contribution of each microorganism from the effects of co-inoculation. Plant height, leaf production, branch emission, shoot dry matter, and root dry matter were selected as integrative indicators of plant vigor and biomass accumulation, providing a practical assessment of whether microbial compatibility translates into measurable agronomic benefits.

The nature of the substrate can play a determining role in maintaining the inoculum. In the present study, a pasteurized, inert substrate (gravel and vermiculite) was used in free-draining pots to avoid interference from native microbiota [34]. Methodologically, this system allows multi-species or multi-genotype cultivation, enabling microbial interactions such as within the *Metarhizium* consortia assessed here [35]. Although the gravel–vermiculite substrate does not reflect the microbial complexity of agricultural soils, its use minimizes background microbial interference during compatibility assessment. Future studies should validate the proposed framework under natural soil conditions, where indigenous microbial communities may influence inoculant performance. Another limitation of the present study is that the proposed framework was validated only under greenhouse conditions. Although this controlled environment was appropriate for the initial assessment of microbial compatibility and plant responses, it did not fully reproduce the environmental variability encountered under field conditions.

The plant growth nutrient solution used in the present study was recommended by the Centre for *Rhizobium* Studies (CRS) and was selected to optimize plant development. This solution prevents chlorine toxicity and maintains iron bioavailability through chelation [34], which is essential for nodule function and overall plant vigor. Although preliminary studies are recommended to test specific biological needs, this combined substrate and nutrient baseline provides a highly reproducible environment for compatibility trials.

In the present methodological framework, plant harvest was strategically performed at 47 DPI (R1 stage) (Figure 2A) to allow sufficient time for fungal establishment while capturing the peak of nodulation. Even under field conditions, initial nodule formation in *G. max* occurs shortly after emergence, and nodulation continuously intensifies until flowering or pod formation [36]. Our findings demonstrated that co-inoculation in *G. max* preserved the integrity of the rhizobia–legume symbiosis, as evidenced by the stability of nodulation (Figure 2F).

Among all evaluated variables, nodulation parameters represent one of the most critical indicators of compatibility between EPF and rhizobia. Since many *Bradyrhizobium* spp. are best known as symbionts that fix nitrogen in legume nodules [37], reductions in nodule formation or nodule biomass can indicate disruption of the symbiotic process. The maintenance of nodulation across all co-inoculated treatments suggests that the presence of *Metarhizium* did not interfere with rhizobial establishment, corroborating the compatibility initially observed in the dual-culture assay.

Root morphology analysis represents a complementary stage of the proposed framework, allowing the detection of functional plant responses that may not be captured by conventional growth and biomass measurements [38]. Fine root number, total root number, and bifurcation frequency were the most informative variables because they reflect the expansion of the absorptive root network. Changes in these traits may represent early plant responses to microbial colonization, even when differences in aboveground growth are not yet evident. In contrast, average root diameter and thick root abundance provided complementary information regarding root structural allocation and responses to nutrient availability. The inclusion of this comprehensive set of root traits therefore enables a more robust characterization of plant–microbe interactions and provides an additional layer of functional screening within the proposed framework.

The increase in fine root number observed for the BR 86 + LCM S04 co-inoculation may indicate a shift in root system architecture toward greater absorptive capacity, since fine roots are primarily responsible for water and nutrient uptake. However, the biological significance of this response should be interpreted with caution, as enhanced fine root production does not necessarily translate into improved plant performance under all growing conditions.

While agronomic variables and nodulation provide information on plant performance and preservation of the rhizobium–legume symbiosis, image-based root phenotyping offers a more detailed assessment of how microbial inoculants influence root system development [39].

To verify EPF establishment and persistence within the experimental system, fungal re-isolation from the substrate was included as a final step of the proposed framework. Substrate samples were collected at the end of the experiment (47 DPI, R1 stage), and both fungal isolates remained cultivable, indicating their persistence during a critical period of soybean development and plant–microbe interaction. Previous studies have demonstrated that *M. anisopliae* LCM S04 can persist for up to five months in switchgrass (*Urochloa brizantha* cv. Marandu) pots under semi-field conditions [40], while *Metarhizium* spp. have been detected for up to one year in strawberry (*Fragaria × ananassa* Duch.) cultivated soils [41]. Although the present assessment was performed relatively soon after inoculation, the methodology can be readily extended to longer monitoring periods through periodic substrate sampling. Although the present study confirms rhizosphere persistence, it does not distinguish between rhizosphere competence and endophytic establishment. Accordingly, future studies incorporating tissue re-isolation or microscopy-based approaches are important to complement the proposed methodology.

The proposed framework can be adapted to evaluate other ecological gains from EPF, such as plant disease mitigation. Previous studies reported that metabolites from various EPF, not limited to *Metarhizium*, suppress necrotrophic phytopathogens and reduce disease incidence via endophytic colonization and immune system priming [16,42,43,44]. For example, a previous study in soybean [45] reported that *Metarhizium* secondary metabolites, such as aurovertins and fungerin, exert antagonistic effects against soil-borne oomycetes like *Phytophthora sojae* and *Aphanomyces cochlioides*. Although investigating disease suppression was beyond the scope of the current study, our sequential protocol, involving in vitro compatibility followed by substrate inoculation, provides a ready-to-use baseline. Researchers can seamlessly integrate phytopathogens into this matrix to evaluate disease severity, tripartite compatibility, or pathogen-induced plant physiological responses, thereby expanding the applicability of this standardized screening platform.

## 4. Materials and Methods

### 4.1. Experimental Location

The in vitro experiments were conducted at the Laboratory of Microbial Control of Arthropods of Veterinary Importance (LCM), located at the Wilhelm Otto Neitz Experimental Station for Parasitological Research (EEPPWON) (22°45′54.9″ S, 43°41′57.2″ W), in the Department of Animal Parasitology (DPA), Institute of Veterinary Medicine, Federal Rural University of Rio de Janeiro (UFRRJ, Seropédica, RJ, Brazil). Greenhouse experiments were carried out at Embrapa Agrobiologia (Seropédica, RJ, Brazil). Plant root system morphological analyses were performed at the Laboratory of Soil Biological Chemistry, Department of Soils, Institute of Agronomy, UFRRJ.

### 4.2. Fungal Strains and Conidial Suspensions

Two fungal strains were used in this study: *Metarhizium anisopliae* LCM S04 [40] and *Metarhizium brunneum* LCM S11. The isolates were obtained from soil samples collected in Rio de Janeiro, RJ, Brazil [40,46]. Both are deposited in the Entomopathogenic Fungi Culture Collection of the Microbial Control Laboratory (CCFELCM/UFRRJ) and in the Filamentous Fungi Culture Collection of the Oswaldo Cruz Institute, Oswaldo Cruz Foundation (Fiocruz) (CCFF/IOC), Rio de Janeiro, RJ, Brazil, under accession numbers IOC 4694 (LCM S04) and IOC 4702 (LCM S11). The selection of these isolates was based on previous evaluations conducted by the research group, in which parameters such as thermotolerance and conidial production capacity were considered.

The fungal strains were cultured on potato-dextrose-agar (PDA) medium (Kasvi, Paraná, PR, Brazil) in polypropylene Petri dishes (90 × 15 mm) and incubated at 25 ± 1 °C and relative humidity (RH) ≥ 80% for 14 days. Conidial suspensions were prepared by adding aerial conidia to sterile water containing 0.01% (*v*/*v*) polyoxyethylene sorbitan monooleate (Tween^®^ 80; Vetec Fine Chemicals Ltda, RJ, Brazil), and concentration was adjusted using a hemocytometer. Fungal viability was assessed by plating 20 µL of 1 × 10^5^ conidia mL^−1^ on PDA, and conidial germination was determined 24 h after incubation at 25 ± 1 °C and RH ≥ 80% using an optical microscope (400×) (ECLIPSE E200; Nikon, Tokyo, Japan). A minimum of 300 conidia were evaluated, and the percentage of germination was calculated. Conidia were considered germinated when the germ tube was visible. The fungal suspensions used in the experiments had a viability of at least 95%.

### 4.3. Bacterial Strains

Two rhizobial strains were used in this study: *Bradyrhizobium diazoefficiens* strain BR 85 (CPAC 7; SEMIA 5080) and *Bradyrhizobium japonicum* strain BR 86 (CPAC 15; SEMIA 5079). The strains were originally isolated in 1985 in Planaltina (15°36′15.2″ S, 47°42′51.7″ W), Federal District, Brazil, in the Cerrado biome [47,48]. Both are deposited at the Embrapa Agrobiologia Johanna Dobereiner Center for Biological Resources (CRB-JD) (Seropédica, RJ, Brazil). The bacterial strains were cultivated in Yeast Mannitol Agar (YMA) medium supplemented with Congo Red (CR) (25 mg L^−1^) (YMA+CR) [49]. The Petri dishes were incubated at 30 ± 1 °C for 12 days.

### 4.4. Co-Inoculant Compatibility Test

The interaction between the fungal isolates *M. anisopliae* LCM S04 and *M. brunneum* LCM S11 and the bacterial strains *B. diazoefficiens* BR 85 and *B. japonicum* BR 86 was evaluated using a dual culture confrontation technique in Petri dishes [50] containing PDA, with minor adaptations [51]. Compatibility was determined based on the radial growth of the fungi. Bacterial colonies, previously cultured in YMA+CR medium, were transferred to plates containing PDA. First, bacteria were inoculated by drawing a continuous line on the surface of the medium, forming a quadrangular arrangement. Then, 20 µL of the fungal suspension (10^7^ conidia mL^−1^) was added to the center (approximately 2 cm away).

The groups consisted of: (I) LCM S04 (*M. anisopliae*) (control); (II) LCM S04 + BR 85 (*B. diazoefficiens*); (III) LCM S04 + BR 86 (*B. japonicum*); (IV) LCM S11 (*M. brunneum*) (control); (V) LCM S11 + BR 85; and (VI) LCM S11 + BR 86. The plates were incubated at 28 ± 1 °C and RH ≥ 80% for 20 days, and radial growth was evaluated at 4, 7, 11, 13, 15, 18 and 20 days after inoculation (DAI) on two perpendicular axes using a millimeter ruler. The average of the two measurements was considered representative of radial growth. The plates were previously identified and marked on the vertical and horizontal axes, maintaining the same direction of measurements throughout the experiment. The experiment was conducted in three independent trials, using new conidial suspensions and bacterial cultures each time. A completely randomized design (CRD) was adopted, with six replicates per treatment, considering each Petri dish as an experimental unit.

The data were first tested for normality of distribution and homoscedasticity of variances using the Shapiro–Wilk test (*p* < 0.05) and Bartlett’s test (*p* < 0.05), respectively. Once these assumptions were confirmed, a two-way Analysis of Variance was applied to observe the isolated and interaction effects. Subsequently, Tukey’s test (*p* < 0.05) was used to compare the groups at each evaluation time, and Regression Analysis was used to assess the behavior of the groups over the studied time periods. Linear or quadratic models were applied, considering both statistical significance and the highest coefficient of determination (R^2^). All analyses were performed using GraphPad Prism 10.4 software.

### 4.5. Plant Inoculation Test

A greenhouse experiment was performed to evaluate the association of soybean (cv. Brasmax Olimpo IPRO) with the strains *M. anisopliae* LCM S04, *M. brunneum* LCM S11, *B. diazoefficiens* BR 85, *B. japonicum* BR 86, their combinations, and uninoculated and nitrogen-fertilized controls. The study assessed the effects of inoculation on vegetative development, with assessment performed at 47 DPI, corresponding to the R1 stage (beginning bloom). Plant growth stages were determined as described in [52]. Seed surface sterilization was performed via immersion in ethanol (70%) for 2 min, followed by immersion in sodium hypochlorite (2%) for 2 min, and finally with eight successive washes with sterile distilled water prior to inoculation.

#### 4.5.1. Bacterial Inoculants

Inoculants of the strains *B. diazoefficiens* BR 85 and *B. japonicum* BR 86 were previously cultured in YMA+CR medium and transferred to test tubes containing 5 mL of BP liquid medium [53] using one colony per tube. The cultures were (1) incubated in an orbital shaker at 30 ± 1 °C and 150 rpm for 72 h (Labinfarma Scientific); (2) transferred to 500 mL Erlenmeyer flasks containing 50 mL of the same medium and incubated under the same conditions for 48 h; and (3) 25 mL of each culture was inoculated into 1000 mL Erlenmeyer flasks containing 250 mL of BP medium, maintaining the temperature and agitation for a further 48 h.

Bacterial growth was monitored by optical density readings at 600 nm (OD_600_) using a UV–visible spectrophotometer (Global Analyzer, Jaboticabal, SP, Brazil) with cultures being used only after reaching OD_600_ ≥ 1.0. At each scaling step, aliquots were plated on YMA+CR medium to verify the purity of the inoculum. Every step of liquid inoculants followed the recommendations of Normative Instruction SDA/MAPA No. 30, of November 12, 2010 [54].

#### 4.5.2. Quantification of Bradyrhizobium Colony-Forming Units (CFU)

To determine the concentration of viable cells, inoculant samples were subjected to serial dilution in sterile saline solution (0.85%). The quantification of viable cells was performed by the surface-spreading plating method at 10^−5^, 10^−6^, and 10^−7^ dilutions, plated in triplicate onto YMA+CR medium. The plates were incubated at 30 ± 1 °C. Evaluation was performed after five to seven days of incubation by selecting plates ranging from 30 to 300 colonies. The results were expressed in colony-forming units per milliliter (CFU mL^−1^) of inoculant.

#### 4.5.3. Plant Growth and Development

The experiment was conducted in a greenhouse under sterile conditions from September to November 2025. The experimental design was a randomized complete block design (RCBD) with ten treatments and seven replications, totaling 70 experimental units (pots). Eight seeds were initially sown per pot, and seedlings were subsequently thinned to two plants per pot. At the time of evaluation, both plants within each pot were analyzed individually for all measured variables, resulting in a total of 140 plant observations. However, because the pot was considered the experimental unit, the values obtained from the two plants within each pot were averaged, and these mean values were used for all statistical analyses (n = 7 pots per treatment).

Gardening pots (3 L) were used as experimental units. The pots were previously washed and internally lined with resistant plastic bags (30 × 40 cm), which were surface-sterilized with 70% (*v*/*v*) ethanol. The pots were filled with a homogenized substrate of gravel and vermiculite (1:1, *v*:*v*) and subsequently sterilized. In the center of each pot, a 20 cm long polyvinyl chloride (PVC) pipe was inserted, sealed at the upper end with a Falcon-type cap, and used as an irrigation and fertigation system. The experimental treatments consisted of: (I) control (CTR); (II) nitrogen control (NIT); (III) BR 85 (*B. diazoefficiens*); (IV) BR 86 (*B. japonicum*); (V) LCM S04 (*M. anisopliae*); (VI) LCM S11 (*M. brunneum*); (VII) LCM S04 + BR 85; (VIII) LCM S04 + BR 86; (IX) LCM S11 + BR 85; and (X) LCM S11 + BR 86.

Irrigation was performed by applying 60 mL of sterile water every three days, or as needed by the plants, as well as the application of the CRS nutrient solution. For the NIT treatment, nitrogen fertilizer (NH_4_NO_3_, 35% N; Química Moderna, São Paulo, SP, Brazil) was diluted in sterile distilled water and applied in seven equal applications (19, 26, 28, 31, 35, 38, and 42 DPI) via the PVC tubing, totaling 4.2 g of NH_4_NO_3_ per pot (0.6 g per pot per application) throughout the experiment.

**Figure 5:**
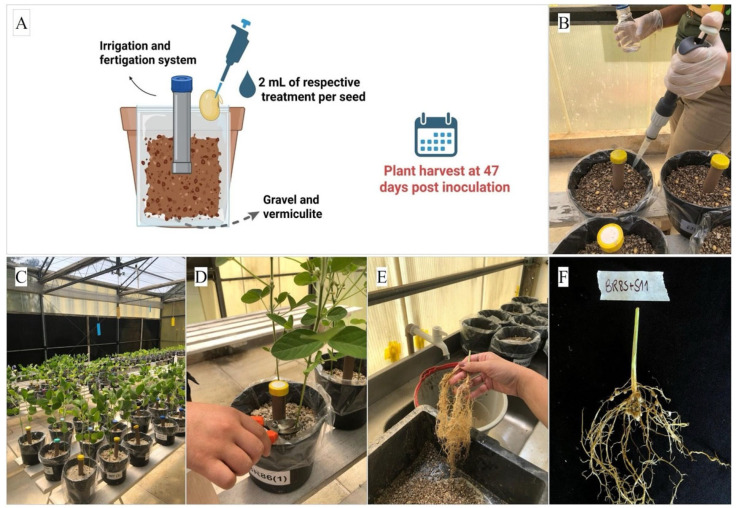
Greenhouse experiment evaluating soybean crop development under inoculation with *Bradyrhizobium* spp. and *Metarhizium* spp. (**A**) Schematic representation of the experimental design; (**B**) Inoculation of microbial suspensions after sowing; (**C**) Plants during experiment conduction; (**D**) Shoot harvesting at the sampling time; (**E**) Root washing for substrate removal; (**F**) Root nodules under co-inoculation.

The experimental procedure followed the methodology described previously [55] (Figure 5). Fungal suspensions (10^7^ conidia mL^−1^) and bacterial inoculants were prepared as described in Section 4.2 and Section 4.5.1, respectively. Inoculation was performed by applying 1 mL of bacterial culture per seed in treatments containing rhizobia + 1 mL of fungal suspension in treatments with *Metarhizium* and 1 mL of Tween 80 solution (0.01%) in the control treatment. To standardize the volume among treatments, those that received only the individual inoculum (bacterial or fungal) as well as the control treatment) additionally received 1 mL of Tween 80 (0.01%), with a final volume of 2 mL seed^−1^ in all treatments. Considering that 8 seeds were sown per experimental unit, the total volume applied corresponded to 16 mL. The seeds were covered with a thin layer of previously washed sterilized sand. Plants were manually watered every two or three days, as needed, and entomological traps were maintained. Thinning was performed at 10 DAS (VE-VC stages, emergence to cotyledon expansion), leaving two plants per pot.

At 47 DPI, plants were harvested and separated into roots and shoots by cutting approximately 1 cm above the ground. Roots were carefully washed under running water and preserved in 30 × 40 cm plastic bags containing a 50% (*v*/*v*) ethanol solution to maintain structural integrity until morphological analysis. The following variables were evaluated: plant height (cm), number of branches (NB) and leaves (NL), shoot dry matter (SDM; g plant^−1^), nodule number (NN), nodule dry matter (NDM; g plant^−1^) and root dry matter (RDM; g plant^−1^).

The height of the plants was measured from the base to the tip of the last true leaf. The root nodules were manually detached and quantified. The aerial part, roots, and nodules were separately packaged in brown paper bags and subjected to drying in a forced-air oven at 60 °C for seven days. The dried material was then weighed to determine the dry mass.

The data were first tested for normality of residuals and homogeneity of variances by using the Shapiro–Wilk (*p* < 0.05) and Bartlett (*p* < 0.05) tests, respectively. The variables number of branches, nodule number and nodule dry matter were subjected to log (x + 0.5) transformation to meet the assumptions of the analysis of variance. The data obtained were subjected to analysis of variance (ANOVA), followed by Tukey’s test for comparison of means, adopting a significance level of 5%. All analyses were performed using R software (version 4.2.2; R Core Team, 2024) in the RStudio environment (version 2025.05.0; Posit Team) using the ExpDes package version 1.2.2 [56].

#### 4.5.4. Root Morphology

Root system morphology was evaluated following a previously described methodology [57], with a total of 140 root samples analyzed. The roots were uniformly arranged in a thin layer of water on a clear acrylic tray (30 cm x 20 cm). Scanning of the plants was performed at 600 dpi (dots per inch) using an Epson Expression 10000XL scanner (Seiko Epson Corporation, Suwa, Nagano, Japan) equipped with an additional light unit (ALU) [58]. The root images were converted into an 8-bit grayscale format for processing. Each image was individually analyzed using the software WinRHIZO Arabidopsis 2012b (Régent Instruments Inc., Quebec City, Canada) for analysis of root characteristics and their quantification [59].

Different root parameters were analyzed and quantified: total number of roots, crossings and bifurcations, root length (mm), root surface area (mm^2^), average root diameter (mm), and root volume (mm^3^). The number of tips within the root classes was also defined and measured by classifying them according to the diameter (d), as follows: fine (<1.5 mm 10^−1^), medium (1.5 < d < 3.0 mm 10^−1^) and thick (d > 3.0 mm 10^−1^).

The averages were tested by Tukey’s test at 5% probability using the Sisvar software. The graphs were created using SigmaPlot software version 5.4 (Build 80).

#### 4.5.5. Fungal Recovery and Qualitative Identification from the Substrate

At the end of the greenhouse experiment (47 DPI), substrate samples were collected from the *Metarhizium*-inoculated pots to verify fungal occurrence. For the non-inoculated, nitrogen-fertilized, and rhizobia-only treatments, samples were randomly collected as negative controls to confirm the absence of *Metarhizium* spp. The substrate was gathered using a sterile spatula from three distinct points within each pot and pooled to form a homogeneous composite sample.

Aliquots of 0.35 g from each composite sample were transferred to 1.5 mL microtubes containing 1 mL of sterile distilled water supplemented with 0.01% Tween 80^®^. The suspensions were vortex-mixed for 1 min and, subsequently, 50 µL aliquots of the suspension were plated onto Chloramphenicol-Tetracycline-Cycloheximide (CTC) selective medium [60] and spread uniformly using a sterile Drigalski loop. The plates were incubated in a growth chamber at 25 ± 1 °C and 80% RH, with evaluations performed between 4 and 14 days after plating.

The presence of *Metarhizium* spp. in the substrate was confirmed by the detection of colonies with characteristic morphology, demonstrating that the fungal inoculants remained recoverable throughout the experimental period.

## 5. Conclusions

This study establishes a methodological framework to evaluate the compatibility and co-inoculation effects of EPF and rhizobia on plant growth and root morphology. By integrating in vitro compatibility assays with detailed greenhouse morphometric analysis, our approach characterized the outcomes of these microbial interactions within the rhizosphere. The proposed methodology proved effective for screening compatible microbial consortia and evaluating their potential effects on root architecture and biomass while ensuring the maintenance of the rhizobia–legume symbiosis. This framework provides a tool for the strategic selection of multi-trophic microbial combinations, offering a standardized pathway to develop sustainable bio-inputs for tropical agriculture.

## Data Availability

The original contributions presented in this study are included in the article/Supplementary Material. Further inquiries can be directed to the corresponding author.

## Supplementary Materials

The following supporting information can be downloaded at: https://www.mdpi.com/article/10.3390/plants15142141/s1. Figure S1: In vitro compatibility assay of isolate LCM S04 (*Metarhizium anisopliae*) grown in dual culture with strains BR 85 (*Bradyrhizobium diazoefficiens*) and BR 86 (*Bradyrhizobium japonicum*) throughout the incubation period. Line (A) represents the control (LCM S04); line (B), LCM S04 + BR 85; and line (C), LCM S04 + BR 86; Figure S2: In vitro compatibility assay of isolate LCM S11 (*Metarhizium brunneum*) grown in dual culture with strains BR 85 (*Bradyrhizobium diazoefficiens*) and BR 86 (*Bradyrhizobium japonicum*) throughout the incubation period. Line (A) represents the control (LCM S11); line (B), LCM S11 + BR 85; and line (C), LCM S11 + BR 86; Figure S3: Re-isolation of *Metarhizium* spp. from substrate samples collected at 47 days post-inoculation (DPI) and cultured on CTC medium to verify fungal persistence in the experimental system. Treatments consisted of: (A) untreated control (CTR); (B) nitrogen-fertilized control (NIT); (C) BR 85 (*Bradyrhizobium diazoefficiens*); (D) BR 86 (*Bradyrhizobium japonicum*); (E) LCM S04 (*Metarhizium anisopliae*); (F) BR 85 + LCM S04; (G) BR 86 + LCM S04; (H) LCM S11 (*Metarhizium brunneum*); (I) BR 85 + LCM S11; and (J) BR 86 + LCM S11.

## References

[1] Iwanicki N.S., Pereira A.A., Botelho A.B.R.Z., Rezende J.M., de Andrade Moral R., Zucchi M.I., Delalibera Júnior I. (2019). Monitoring of the field application of *Metarhizium anisopliae* in Brazil revealed high molecular diversity of *Metarhizium* spp. in insects, soil and sugarcane roots. Sci. Rep..

[2] Mascarin G.M., Jaronski S.T. (2016). The production and uses of *Beauveria bassiana* as a microbial insecticide. World J. Microbiol. Biotechnol..

[3] Mascarin G.M., Golo P.S., de Souza Ribeiro-Silva C., Quintela E.D., Jaronski S.T., Delalibera Júnior I. (2024). Advances in submerged liquid fermentation and formulation of entomopathogenic fungi. Appl. Microbiol. Biotechnol..

[4] Behie S.W., Jones S.J., Bidochka M.J. (2015). Plant tissue localization of the endophytic insect pathogenic fungi *Metarhizium* and *Beauveria*. Fungal Ecol..

[5] Quesada-Moraga E. (2020). Entomopathogenic fungi as endophytes: Their broader contribution to IPM and crop production. Biocontrol Sci. Technol..

[6] Stone L.B.L., Bidochka M.J. (2020). The multifunctional lifestyles of *Metarhizium*: Evolution and applications. Appl. Microbiol. Biotechnol..

[7] Mesquita E., Hu S., Lima T.B., Golo P.S., Bidochka M.J. (2023). Utilization of *Metarhizium* as an insect biocontrol agent and a plant bioinoculant with special reference to Brazil. Front. Fungal Biol..

[8] Bamisile B.S., Afolabi O.G., Siddiqui J.A., Xu Y. (2023). Endophytic insect pathogenic fungi-host plant-herbivore mutualism: Elucidating the mechanisms involved in the tripartite interactions. World J. Microbiol. Biotechnol..

[9] Aravinthraju K., Shanthi M., Murugan M., Srinivasan R., Maxwell L.A., Manikanda Boopathi N., Anandham R. (2024). Endophytic entomopathogenic fungi: Their role in enhancing plant resistance, managing insect pests, and synergy with management routines. J. Fungi.

[10] Canassa F., Esteca F.C.N., Moral R.A., Meyling N.V., Klingen I., Delalibera Júnior I. (2020). Root inoculation of strawberry with the entomopathogenic fungi *Metarhizium robertsii* and *Beauveria bassiana* reduces incidence of the twospotted spider mite and selected insect pests and plant diseases in the field. J. Pest Sci..

[11] Holz S., D’Alessandro C.P., Maximo H.J., Nascimento de Souza P.H., Raruang Y., Demétrio C.G.B., Chen Z.Y., Delalibera Júnior I. (2023). The potential of using *Metarhizium anisopliae* and *Metarhizium humberi* to control the Asian soybean rust caused by *Phakopsora pachyrhizi*. Biocontrol Sci. Technol..

[12] Khan A.L., Hamayun M., Khan S.A., Kang S.M., Shinwari Z.K., Kamran M., Rehman S., Kim J.G., Kang S.M., Lee I.J. (2012). Pure culture of *Metarhizium anisopliae* LHL07 reprograms soybean to higher growth and mitigates salt stress. World J. Microbiol. Biotechnol..

[13] Golo P.S., Gardner D.R., Grilghagen N.M., Takatsuka J., Zimmer C.R., Castilho A.M.C., Bittencourt V.R.E.P., Roberts D.W. (2014). Production of destruxins from *Metarhizium* spp. fungi in artificial medium and in endophytically colonized cowpea plants. PLoS ONE.

[14] Hu S., Bidochka M.J. (2021). Root colonization by endophytic insect-pathogenic fungi. J. Appl. Microbiol..

[15] Liao X., O’Brien T.R., Fang W., St. Leger R.J. (2014). The plant beneficial effects of *Metarhizium* species correlate with their association with roots. Appl. Microbiol. Biotechnol..

[16] Sarven M.S., Hao Q., Deng J., Yang F., Wang G., Xiao Y., Xiao X. (2020). Biological control of tomato gray mold caused by *Botrytis cinerea* with the entomopathogenic fungus *Metarhizium anisopliae*. Pathogens.

[17] Russo M.L., Pelizza S.A., Cabello M.N., Stenglein S.A., Scorsetti A.C. (2015). Endophytic colonisation of tobacco, corn, wheat and soybeans by the fungal entomopathogen *Beauveria bassiana* (Ascomycota, Hypocreales). Biocontrol Sci. Technol..

[18] Ahmad I., Del Mar Jiménez-Gasco M., Luthe D.S., Barbercheck M.E. (2020). Systemic colonization by *Metarhizium robertsii* enhances cover crop growth. J. Fungi.

[19] Hu S., Mojahid M.S., Bidochka M.J. (2023). Root colonization of industrial hemp (*Cannabis sativa* L.) by the endophytic fungi *Metarhizium* and *Pochonia* improves growth. Ind. Crops Prod..

[20] Bais H.P., Weir T.L., Perry L.G., Gilroy S., Vivanco J.M. (2006). The role of root exudates in rhizosphere interactions with plants and other organisms. Annu. Rev. Plant Biol..

[21] Singh B.K., Millard P., Whiteley A.S., Murrell J.C. (2004). Unravelling rhizosphere-microbial interactions: Opportunities and limitations. Trends Microbiol..

[22] Seixas C.D.S., Neumaier N., Balbinot Junior A.A., Krzyzanowski F.C., Leite R.M.V.B.C. (2020). Tecnologias de Produção de Soja.

[23] Hungria M., Campo R.J., Mendes I.C., Graham P.H., Singh R.P., Shankar N., Jaiwal P.K. (2006). Contribution of Biological Nitrogen Fixation to the N Nutrition of Grain Crops in the Tropics: The Success of Soybean (*Glycine max* L. Merr.) in South America. Nitrogen Nutrition and Sustainable Plant Productivity.

[24] Hungria M., Mendes I.C., de Bruijn F.J. (2015). Nitrogen Fixation with Soybean: The Perfect Symbiosis?. Biological Nitrogen Fixation.

[25] Chibeba A.M., Kyei-Boahen S., Guimarães M.F., Nogueira M.A., Hungria M. (2018). Feasibility of transference of inoculation-related technologies: A case study of evaluation of soybean rhizobial strains under the agro-climatic conditions of Brazil and Mozambique. Agric. Ecosyst. Environ..

[26] Fukami J., De La Osa C., Ollero F.J., Megías M., Hungria M. (2018). Co-inoculation of maize with *Azospirillum brasilense* and *Rhizobium tropici* as a strategy to mitigate salinity stress. Funct. Plant Biol..

[27] Ahsan S.M., Injamum-Ul-Hoque M., Das A.K., El-Sharnouby M.E., El-Sheery M.I., Alorfi H.S., Ali E.F., Ahmed A.F., Sharaf M. (2024). Plant–Entomopathogenic Fungi Interaction: Recent Progress and Future Prospects on Endophytism-Mediated Growth Promotion and Biocontrol. Plants.

[28] Rasool S., Jensen B., Roitsch T.G., Meyling N.V. (2024). Enzyme Regulation Patterns in Fungal Inoculated Wheat May Reflect Resistance and Tolerance Towards an Insect Herbivore. J. Plant Physiol..

[29] Iglesias I., Beyhaut E., Rivas-Franco F. (2026). Native multifunctional *Metarhizium* spp. delivered via seed coatings suppress soil-borne phytopathogens and preserve rhizobial symbiosis in soybean. Biocontrol Sci. Technol..

[30] Blum B.J. Concepts and strategies for a successful product development: The industry’s development concept. Proceedings of the COST Action 850 Conference.

[31] Vega F.E., Meyling N.V., Luangsa-ard J.J., Blackwell M., Vega F.E., Kaya H.K. (2012). Fungal entomopathogens. Insect Pathology.

[32] Behie S.W., Bidochka M.J. (2014). Ubiquity of insect-derived nitrogen transfer to plants by endophytic insect-pathogenic fungi: An additional branch of the soil nitrogen cycle. Appl. Environ. Microbiol..

[33] Westphal K.R., Rodrigues A., Larsen T.O., Frisvad J.C. (2021). The effects of different potato dextrose agar media on secondary metabolite production in *Fusarium*. Int. J. Food Microbiol..

[34] Howieson J.G., Dilworth M.J. (2016). Working with Rhizobia.

[35] Howieson J.G., Loi A., Carr S.J. (1995). *Biserrula pelecinus* L.—A legume pasture species with potential for acid, duplex soils which is nodulated by unique root-nodule bacteria. Aust. J. Agric. Res..

[36] Hungria M., Vargas A.T., Campo R.J. (1997). A Inoculação da Soja.

[37] Terra L.A., Klepa M.S., Nogueira M.A., Hungria M. (2025). Pangenome analysis indicates evolutionary origins and genetic diversity: Emphasis on the role of nodulation in symbiotic *Bradyrhizobium*. Front. Plant Sci..

[38] Stahlhut K.N., Neupert D.G., Laing J.E., Witt L.J., Bauer J.T. (2024). Measuring leaf and root functional traits uncovers multidimensionality of plant responses to arbuscular mycorrhizal fungi. Am. J. Bot..

[39] Islam M.S., Ghimire A., Lay L., Khan W., Lee J.-D., Song Q., Jo H., Kim Y. (2024). Identification of quantitative trait loci controlling root morphological traits in an interspecific soybean population using 2D imagery data. Int. J. Mol. Sci..

[40] Mesquita E., Marciano A.F., Corval A.R.C., Mascarin G.M., Faria M., Delalibera I., Perinotto W.M.S., Bittencourt V.R.E.P., Gôlo P.S. (2020). Efficacy of a native isolate of the entomopathogenic fungus *Metarhizium anisopliae* against larval tick outbreaks under semifield conditions. BioControl.

[41] Castro T., Mayerhofer J., Enkerli J., Eilenberg J., Meyling N.V., Moral R.A., Delalibera I. (2016). Persistence of Brazilian isolates of the entomopathogenic fungi *Metarhizium anisopliae* and *M. robertsii* in strawberry crop soil after soil drench application. Agric. Ecosyst. Environ..

[42] Barelli L., Waller A.S., Behie S.W., Bidochka M.J. (2020). Plant microbiome analysis after *Metarhizium* amendment reveals increases in abundance of plant growth-promoting organisms and maintenance of disease-suppressive soil. PLoS ONE.

[43] Barra-Bucarei L., Iglesias A.F., Torres C.P., Souza B., Marucci R.C., Vásquez L.L. (2019). Entomopathogenic Fungi. Natural Enemies of Insect Pests in Neotropical Agroecosystems.

[44] Hao Q., Albaghdady D.M.D., Xiao Y., Xiao X., Mo C., Tian T., Tang J., Chen L. (2021). Endophytic *Metarhizium anisopliae* is a potential biocontrol agent against wheat *Fusarium* head blight caused by *Fusarium graminearum*. J. Plant Pathol..

[45] Putri S.P., Ishido K., Kinoshita H., Kitani S., Ihara F., Sakihama Y., Igarashi Y., Nihira T. (2014). Production of antioomycete compounds active against the phytopathogens *Phytophthora sojae* and *Aphanomyces cochlioides* by clavicipitoid entomopathogenic fungi. J. Biosci. Bioeng..

[46] Corrêa T.A., Santos F.S., Camargo M.G., Quinelato S., Bittencourt V.R.E.P., Golo P.S. (2022). Comparison of Methods for Isolating Entomopathogenic Fungi from Soil Samples. J. Vis. Exp..

[47] Menna P., Hungria M., Barcellos F.G., Bangel E.V., Hess P.N., Martínez-Romero E. (2006). Molecular phylogeny based on the 16S rRNA gene of elite rhizobial strains used in Brazilian commercial inoculants. Syst. Appl. Microbiol..

[48] Siqueira A.F., Ormeño-Orrillo E., Souza R.C., Rodrigues E.P., Almeida L.G., Barcellos F.G., Batista J.S., Nakatani A.S., Martínez-Romero E., Vasconcelos A.T. (2014). Comparative genomics of *Bradyrhizobium japonicum* CPAC 15 and *Bradyrhizobium diazoefficiens* CPAC 7: Elite model strains for understanding symbiotic performance with soybean. BMC Genom..

[49] Vincent J.M. (1970). A Manual for the Practical Study of Root-Nodule Bacteria.

[50] Mariano R.L.R. (1993). Métodos de seleção “in vitro” para controle microbiológico. Rev. Anu. Patol. Plantas.

[51] Fernandes M.F.R., Ribeiro T.G., Rouws J.R., Soares L.H.B., Zilli J.É. (2021). Biotechnological potential of bacteria from genera *Bacillus*, *Paraburkholderia* and *Pseudomonas* to control seed fungal pathogens. Braz. J. Microbiol..

[52] Fehr W.R., Caviness C.E. (1977). Stages of Soybean Development.

[53] Scheidt W., Pedroza I.C.P.S., Fontana J., Meleiro L.A.C., Soares L.H.B., Reis V.M. (2020). Optimization of culture medium and growth conditions of the plant growth-promoting bacterium *Herbaspirillum seropedicae* BR11417 for its use as an agricultural inoculant using response surface methodology (RSM). Plant Soil.

[54] Ministério da Agricultura, Pecuária e Abastecimento (2010). Instrução Normativa SDA nº 30, de 12 de novembro de 2010. Estabelece os Métodos Oficiais para Análise de Inoculantes, Sua Contagem, Identificação e Análise de Pureza.

[55] Yates R.J., Howieson J.G., Hungria M., Bala A., O’Hara G.W., Terpolilli J.J., Howieson J.G., Dilworth M.J. (2016). Authentication of rhizobia and assessment of the legume symbiosis in controlled plant growth systems. Working with Rhizobia.

[56] Ferreira E.B., Cavalcanti P.P., Nogueira D.A. (2014). ExpDes: An R package for ANOVA and experimental designs. Appl. Math..

[57] Tavares O.C.H. (2014). Efeito dos Ácidos Húmicos Sobre as H^+^-ATPase, Transportadores de N-NO_3_ e N-NH_4_^+^, e Sobre o Crescimento em Arroz. Ph.D. Thesis.

[58] Tavares O.C.H., Santos L.A., Filho D.F., Ferreira L.M., García A.C., Castro T.A.V.T., Zonta E., Pereira M.G., Fernandes M.S. (2021). Response Surface Modeling of Humic Acid Stimulation of the Rice (*Oryza sativa* L.) Root System. Arch. Agron. Soil Sci..

[59] Arsenault J.L., Pouleur S., Messier C., Guay R. (1995). WinRHIZO™, a root-measuring system with a unique overlap correction method. HortScience.

[60] Fernandes E.K.K., Keyser C.A., Rangel D.E.N., Foster R.N., Roberts D.W. (2010). CTC medium: A novel dodine-free selective medium for isolating entomopathogenic fungi, especially *Metarhizium acridum*, from soil. Biol. Control.

