# Supplementary material for: Methodological Framework to Evaluate Entomopathogenic Fungi and Rhizobial Co-Inoculation Effects on Plant Growth and Root Morphology
Source: Plants (Basel). 2026 Jul 10;15(14):2141. doi: 10.3390/plants15142141 (PMC13415070; doi:10.3390/plants15142141)

Figure S1: *In vitro* compatibility assay of isolate LCM S04 (*Metarhizium anisopliae*) grown in dual culture with strains BR 85 (*Bradyrhizobium diazoefficiens*) and BR 86 (*Bradyrhizobium japonicum*) throughout the incubation period. Line (A) represents the control (LCM S04); line (B), LCM S04 + BR 85; and line (C), LCM S04 + BR 86.

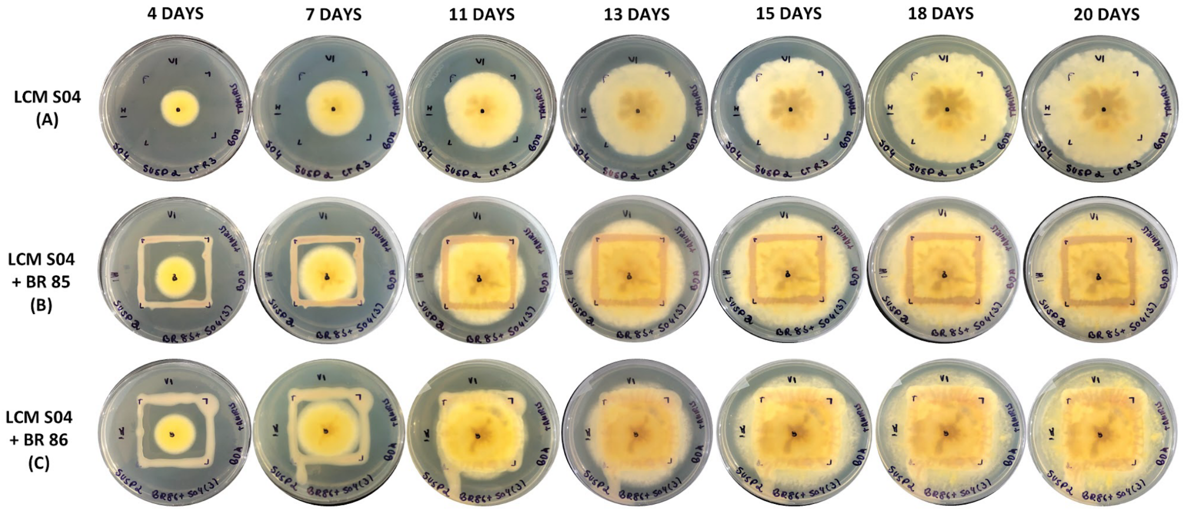

Figure S2: *In vitro* compatibility assay of isolate LCM S11 (*Metarhizium brunneum*) grown in dual culture with strains BR 85 (*Bradyrhizobium diazoefficiens*) and BR 86 (*Bradyrhizobium japonicum*) throughout the incubation period. Line (A) represents the control (LCM S11); line (B), LCM S11 + BR 85; and line (C), LCM S11 + BR 86.

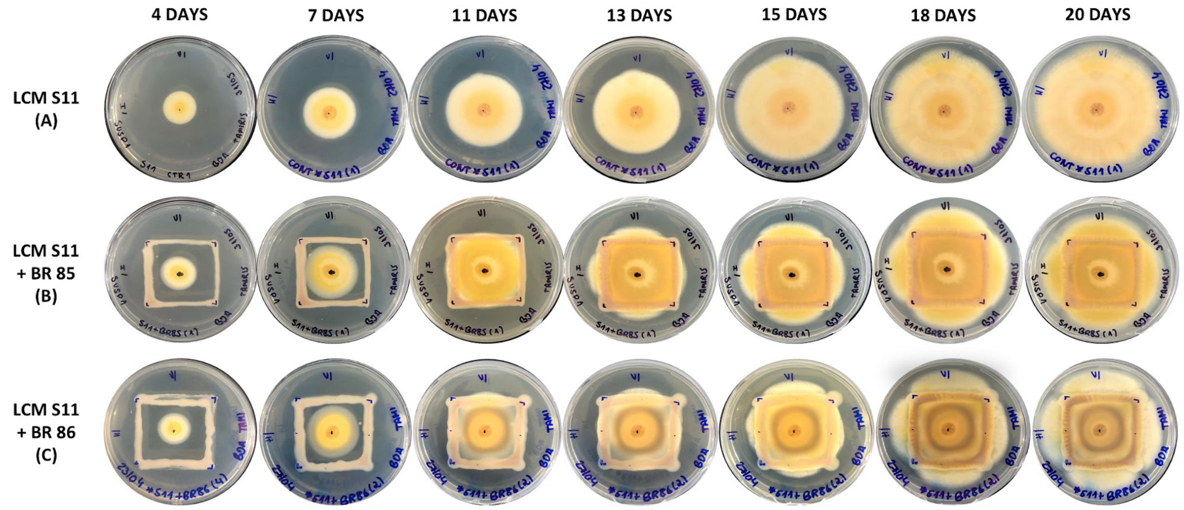

Figure S3: Re-isolation of *Metarhizium* spp. from substrate samples collected at 47 days post-inoculation (DPI) and cultured on CTC medium to verify fungal persistence in the experimental system. Treatments consisted of: (A) untreated control (CTR); (B) nitrogen-fertilized control (NIT); (C) BR 85 (*Bradyrhizobium diazoefficiens*); (D) BR 86 (*Bradyrhizobium japonicum*); (E) LCM S04 (*Metarhizium anisopliae*); (F) BR 85 + LCM S04; (G) BR 86 + LCM S04; (H) LCM S11 (*Metarhizium brunneum*); (I) BR 85 + LCM S11; and (J) BR 86 + LCM S11.

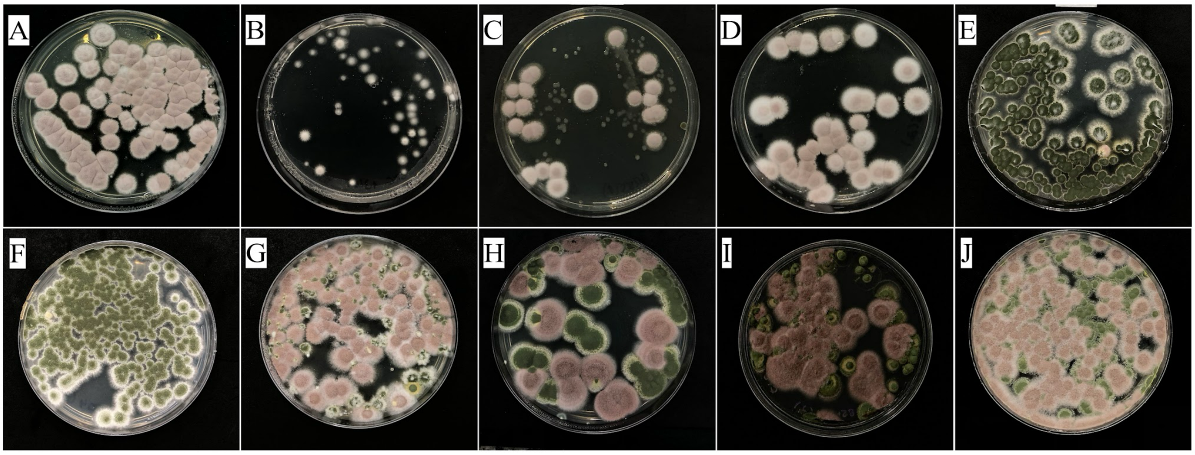

Supplement: Supplementary file 1 [file plants-15-02141-s001.zip › plants-4401245-supplementary.pdf]
